# Clinical evaluation of final impressions from three-dimensional printed custom trays

**DOI:** 10.1038/s41598-017-14005-8

**Published:** 2017-11-02

**Authors:** Yuchun Sun, Hu Chen, Hong Li, Kehui Deng, Tian Zhao, Yong Wang, Yongsheng Zhou

**Affiliations:** 1 Center of Digital Dentistry, Peking University School and Hospital of Stomatology, 22 Zhongguancun Avenue South, Haidian District, Beijing, 100081 People’s Republic of China; 2 Peking University Hospital of Stomatology First Clinical Division, 37A Xishiku Street, Xicheng District, Beijing, 100034 People’s Republic of China; 3 Department of Prosthodontics, Peking University School and Hospital of Stomatology, 22 Zhongguancun Avenue South, Haidian District, Beijing, 100081 People’s Republic of China; 4 National Engineering Laboratory for Digital and Material Technology of Stomatology, Beijing, China; 5 Research Center of Engineering and Technology for Digital Dentistry, Ministry of Health, Beijing, China; 6 Beijing Key Laboratory of Digital Stomatology, Beijing, China

## Abstract

This study aimed to evaluate the quality of the final impressions taken by three-dimensional printed custom trays for edentulous patients. Custom trays were designed with or without saddle-shaped tissue stops and fabricated by three-dimensional printing techniques. Manually made trays with photocurable materials were produced as controls. Both 3D printed custom trays and manually made ones were used to take impressions from edentulous patients. After 3D scanning of the final impression, the impression materials were removed, thus the underneath tray surfaces were able to be scanned, allowing the thickness of the impression materials to be measured. Final impressions obtained by pre-border-molded 3D printed trays were scanned as references, to which the flange extension deviations and morphology deviations of the impressions taken by both 3D printed trays and manually made ones were calculated. The results showed that (1) impressions from 3D printed custom trays had better thickness distribution than that of manually made ones; (2) impression morphology deviations in non-marginal area were neither statistic different between 3D printed trays and manually made trays, nor between trays with and without tissue stops; and (3) final impressions taken by custom trays without pre-border-molding were tended to have insufficient flange extensions.

## Introduction

Taking an accurate edentulous impression is the first step in complete denture restoration and is key to ensuring that the complete dentures will have good support, retention, and stabilization functions^1^. The two-step technique with the aid of custom tray is helpful for obtaining accurate impression morphology and moderate flange extension, compared to the one-step technique with a stock tray^2–4^. The two-step technique involves using primary and final impressions^5,6^. First, the primary impression is constructed from a stock tray with an impression material. Second, the primary plaster model is used to fabricate the custom tray with which the final impression is obtained. The traditional process of manufacturing the custom tray is very complicated, and it is difficult to ensure the wanted space throughout the tray to accommodate the final impression materials^7–9^. In a previous study, this research group developed a digital method of manufacturing three-dimensional (3D)-printed custom trays for edentulous patients by using fused deposition modelling (FDM) technology, which has reduced doctors’ manual operation time. Moreover, this group conducted an *in vitro* experiment to prove that the space for the final impression material in a digitally made custom tray is more accurate than that in the manually made tray^10^. However, its clinical implications need to be evaluated.

Only the correct placement of the tray can hold the final impression material with the wanted thickness, which is important for accurate final working models^9,11^. The use of a tissue stop helps to place the tray correctly. A tissue stop can get support from the primary stress-bearing area, effectively maintaining its stability in three dimensions when the tray is seated^12–15^. Further, a tissue stop helps to seat the tray precisely, although its influence on an impression’s accuracy needs to be fully evaluated because the tissue stop itself may put pressure on tissues in the contact area.

Deviation also occurs because of the deformability of edentulous patients’ soft tissue, even if the same trays and impression materials are adopted and the impressions are obtained repeatedly in the same manner^16–18^. There is no definite standard for obtaining the most accurate impression. The two-step impression technique using a pre-border-molded custom tray (with border-molding wax or compound) seems reasonably reliable in achieving a good extension flange^19,20^. A relatively high accuracy of the impression can be expected because the molded border wax or compound offers a ‘marginal support effect’ (i.e. ‘marginal stop effect’), which is helpful in guiding the placement of custom trays and maintaining the space for final impression materials. Therefore, in this study, the impressions obtained from the pre-border-molded custom trays were set as the ‘gold standard’ to evaluate impression accuracy and the extension flange of 3D printed trays. However, the time-consuming and technique-sensitive pre-border molding process is bothersome for operators to perform. In this study, custom trays designed with or without a tissue stop and produced by high precise 3D printing technology were aimed to improve the impression quality and simplify the procedure. Of particular interest was determining which type of custom tray without a pre-border mold could achieve a better-quality impression (using the impression taken from a pre-border-molded custom tray as the reference).

## Materials and Methods

This study was approved by the bioethics committee of Peking University School and Hospital of Stomatology (Beijing, China; approval number, PKUSSIRB-201627042). The procedures and risks involved with participating in this study were discussed with the volunteers, and written informed consent was obtained from each included participant. The methods were conducted in accordance with the approved guidelines.

Seven edentulous patients from the Department of Prosthodontics of Peking University School of Stomatology in 2016 were enrolled. They had undergone complete extraction of teeth 3 months prior without any serious systemic diseases, non-heavily absorbed alveolar bones (i.e., Atwood levels I–III), and no apparent defects in the mandible and maxilla. Moreover, they had healthy oral mucosa and no hyperactive pharyngeal reflex.

### 3D printing of the custom trays

**Figure 1:**
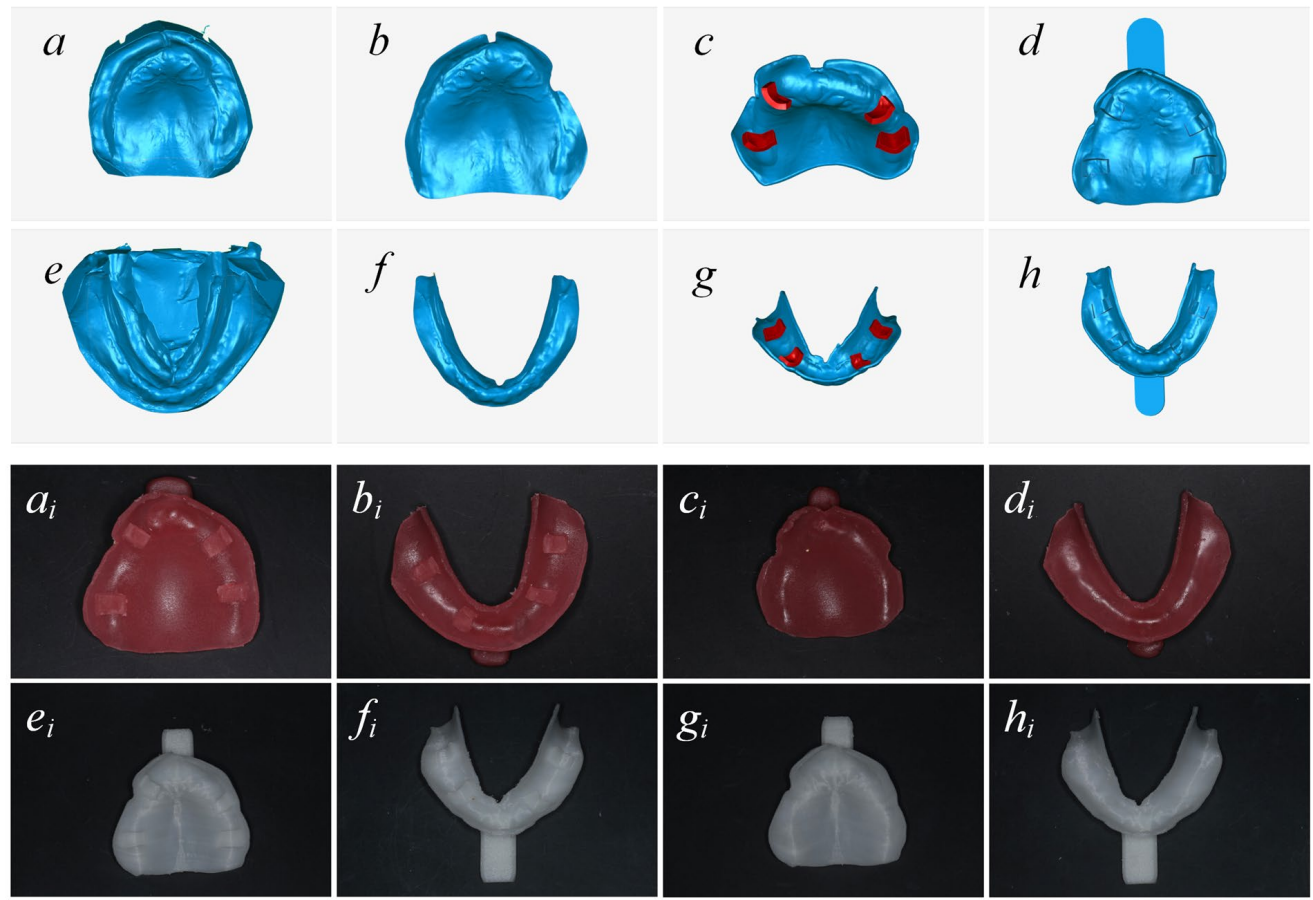
Design process of the digital 3DP tray (**a**–**d** maxilla; **e**–**h** mandible). (**a** and **e**) The scanned data of the primary impression. (**b** and **f**) The impression is trimmed to the appropriate range. (**c** and **g**) The main part of the tray and the tissue stop. (**d** and **h**) A handle is added to the tray. The finished trays (**a**_**i**_ **–d**_**i**_ manual tray; **e**_**i**_ **–h**_**i**_ digital tray). (**a**_**i**_ and **e**_**i**_) A maxillary tray with a tissue stop. (**b**_**i**_ and **f**_**i**_) A mandibular tray with a tissue stop. (**c**_**i**_ and **g**_**i**_) A maxillary tray without a tissue stop. (**d**_**i**_ and **h**_**i**_) A mandibular tray without a tissue stop.

A dental model scanner (Activity 880; Smart Optics, Bochum, Germany) was used to scan the tissue surface of the primary model. The scanned data were imported to custom tray design software (SV Individual Tray; Hoteamsoft Co., Ltd, Jinan, China) to design the body of the tray, and imported to reverse engineering software (Geomagic Studio12.0; Raindrop, Morrisville, NC, USA) to design the tissue stop; After the scanned data were imported (Fig. 1a), the boundary line of the tray was extracted, and unnecessary data were eliminated (Fig. 1b). A 2-mm offset along the surface normal direction of the inside surface of the tray was created, while reserving an wanted 3D space for the impression material. A 2-mm shell feature was then created from the offset surface, thereby forming the body of the virtual tray. Rectangles 5–6 mm long and 4–5 mm wide were chosen at the alveolar ridge crest of the bilateral canines and first molar areas. They were evenly thick by 2 mm in the normal direction, forming a tissue stop and fused with the tray body (Fig. 1c). A predesigned three-dimensional modality handle was added, thereby completing the design of a tray with tissue stop (i.e. 3DPS tray; Fig. 1d).

A tray without a tissue stop (i.e. 3DP tray) was created using the aforementioned method, while the tissue stop design step was skipped.

The designed data was imported to an FDM three-dimensional printer (Lingtong II, SHINO Tech, Beijing, China) to fabricate trays using polylactic acid as the raw material (Fig. 1e_i_–1h_i_).

### Manual manufacturing method for the custom trays

By determining the borderline of the tray in the primary cast, 2 mm of soft wax was paved evenly on the original model, and trimmed to the border line of the tray. After it cooled and hardened, light-cure composite resins were paved on it, and a handle was added. This process was followed by trimming, polishing, and cleaning. After light curing for 10 minutes, the manual tray without a tissue stop (i.e. Manu tray) was manufactured (Fig. 1c_i_ and d_i_).

During the manufacture of the custom tray with a tissue stop (i.e. ManuS tray), part of the wax at the alveolar ridge crest of the bilateral canine and first molar area in the primary impression was removed. A 5- to 6-mm long and 4- to 5-mm wide rectangular ‘window’ was thus formed. The filled photo-curing resin in the window formed the tissue stop after light curing (Fig. 1a_i_ and b_i_)

### Taking the final impression

Each of the four trays (i.e. 3DP tray, 3DPS tray, Manu tray and ManuS tray) were placed in the same patients’ mouth until they fit well. The same doctor then obtained a final impression from the edentulous patients. Although the silicone impression material is more precise and reliable than alginate material, it is not used for edentulous patients in this study because of its expense and long curing time. The final impression was obtained directly by the alginate impression materials with both active and passive functional molding techniques, whereas the custom tray was not pre-border-molded with any border-molding materials. After the doctor made each impression, the patient took a 20-minute break, which allowed time for the pressed and deformed soft tissues to recover. A 5-year experienced prosthodontic dentist performed the experimental operations in this study.

**Figure 2:**
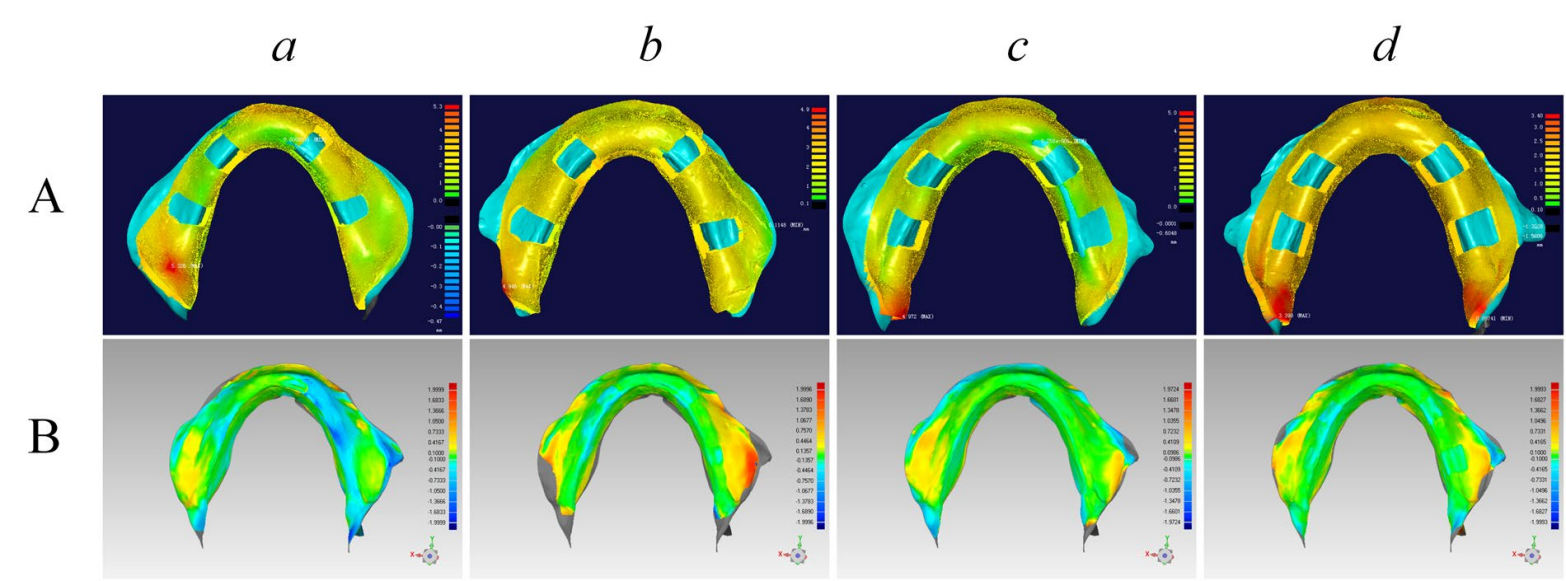
Analysis of the thickness of the final impression material (**A**) and the three-dimensional deviation of the impression surface. (**a**) A manual tray without a tissue stop; (**b**) A manual tray with a tissue stop; (**c**) A three-dimensional (3D)-printed tray without a tissue stop; (**d**) A 3D-printed tray with a tissue stop.

To evaluate the accuracy and flange extension of the impressions acquired with the four types of custom trays, a reference impression for each patient needed to be obtained by a reliable method that used a pre-border-molded custom tray. To this end, a digital 3DP tray was created for each patient, and the trays were pre-border-molded inside the mouth using border molding wax (ISO Functional; GC Company, Tokyo, Japan). The final impression was taken by alginate impression materials, with both active and passive functional molding techniques. Two volunteers could not bear the long time required for the border-molding and impression-taking procedure. However, five patients finished the reference impression-taking. Three-dimensional scanning was immediately applied to the final impressions. The scanned data were used to evaluate the precision of the four trays (Fig. 2b)

### Measurement of the impression material thickness

The final impression was placed into a dental model scanner for the 3D scan. The custom trays and the impression material on them were fixed on the scanner’s platform with hot melt glue. The surfaces of the impressions were scanned. The impression materials on the trays were thereafter carefully removed without taking the trays off the platform. The surface of the tray was then scanned. The thickness of the final impression material was measured as the distance between the impression surface and the underneath tray surface. The scanned data were processed in Geomagic Studio software. For the 3DPS tray and ManuS tray, the area where tissue stop lay was trimmed. To ensure the uniformity of the testing data, areas with the same size and location were trimmed in the 3DP tray and Manu tray (Fig. 2A). The rest of the point-cloud data were screened and simplified with a uniform interval of 2 mm, thereby obtaining the screened point (the quantity is *n*). The distance *x*_*i*_ from each point to the triangular meshing surface of the impression was measured in 3D reverse engineering software (Imageware 13.0; EDS, Pottsville, PA, USA). The final data were analysed using SPSS 17 (IBM, USA), and the power was calculated by PASS 11 (NCSS, LLC.; Kaysville, UT, USA).

By subtracting 2 mm from the distance value of each point to the impression surface, the deviation value of each point distance was obtained. The average value of the absolute deviation value was then calculated, as follows:

$$d=\frac{\sum _{1}^{{\boldsymbol{n}}}{\boldsymbol{ABS}}({{\boldsymbol{x}}}_{{\boldsymbol{i}}}-2)}{{\boldsymbol{n}}}$$

in which *d* is the error of the impression thickness of each tray. The errors in the Manu tray, ManuS tray, 3DP tray, and 3DPS tray was denoted by ${d}_{{\rm{Manu}}}$, ${d}_{{\rm{ManuS}}}$, ${d}_{{\rm{3DP}}}$ and ${d}_{3{\rm{DPS}}}$, respectively. The errors of four trays in the same patient were matched and tested in SPSS for paired Student’s *t* test (${d}_{{\rm{Manu}}}-{d}_{{\rm{ManuS}}}$, ${d}_{3{\rm{DP}}}-{d}_{3{\rm{DPS}}}$, ${d}_{{\rm{Manu}}}-{d}_{3{\rm{DP}}}$ and ${d}_{{\rm{ManuS}}}-{d}_{3{\rm{DPS}}}$). The accuracy of the impression material thickness obtained from the four trays was compared. The significance value was set at P < 0.05.

### Evaluation of the accuracy of the final impression surface and the extension flange

The final impressions of four trays were matched with the reference data (using Geomagic Studio [Raindrop]) with the marginal area trimmed. The data points of the final impression were screened and simplified with a uniform interval of 0.5 mm. The distance between each point of the simplified point clouds and the triangular meshing surface of the reference impression was then calculated using Imageware (EDS). The average value, *E*, of the point distances indicated the deviation of the tissue surface of the final impression. The three-dimensional deviations of the final impressions from the four different trays of the same patient were obtained and analysed using paired Student’s *t* test in SPSS (i.e. ${E}_{{\rm{Manu}}}-{E}_{{\rm{ManuS}}}$, ${E}_{3{\rm{DP}}}-{E}_{3{\rm{DPS}}}$, ${E}_{{\rm{Manu}}}-{E}_{3{\rm{DP}}}\,$ and ${E}_{{\rm{ManuS}}}-{E}_{3{\rm{DPS}}}$). The accuracy of the three-dimensional morphology of the impression surface in four trays was compared. The significance value was set at P < 0.05.

To evaluate the extension flange of the impressions, we imported the well-matched data of the final impressions of the four trays and the reference data to the three-dimensional measuring and analysing software (Geomagic Qualify 12; Raindrop). As shown in Fig. 4a and b, the distance between the borders of the impressions and the reference impressions were obtained by setting a profile along the direction of the canine teeth and the first molars buccolingual perpendicular to the dento-occlusal plane. A positive value indicated that the flange extension of the measured impression was smaller than that of the reference and a negative value indicate larger extension.

**Figure 3:**
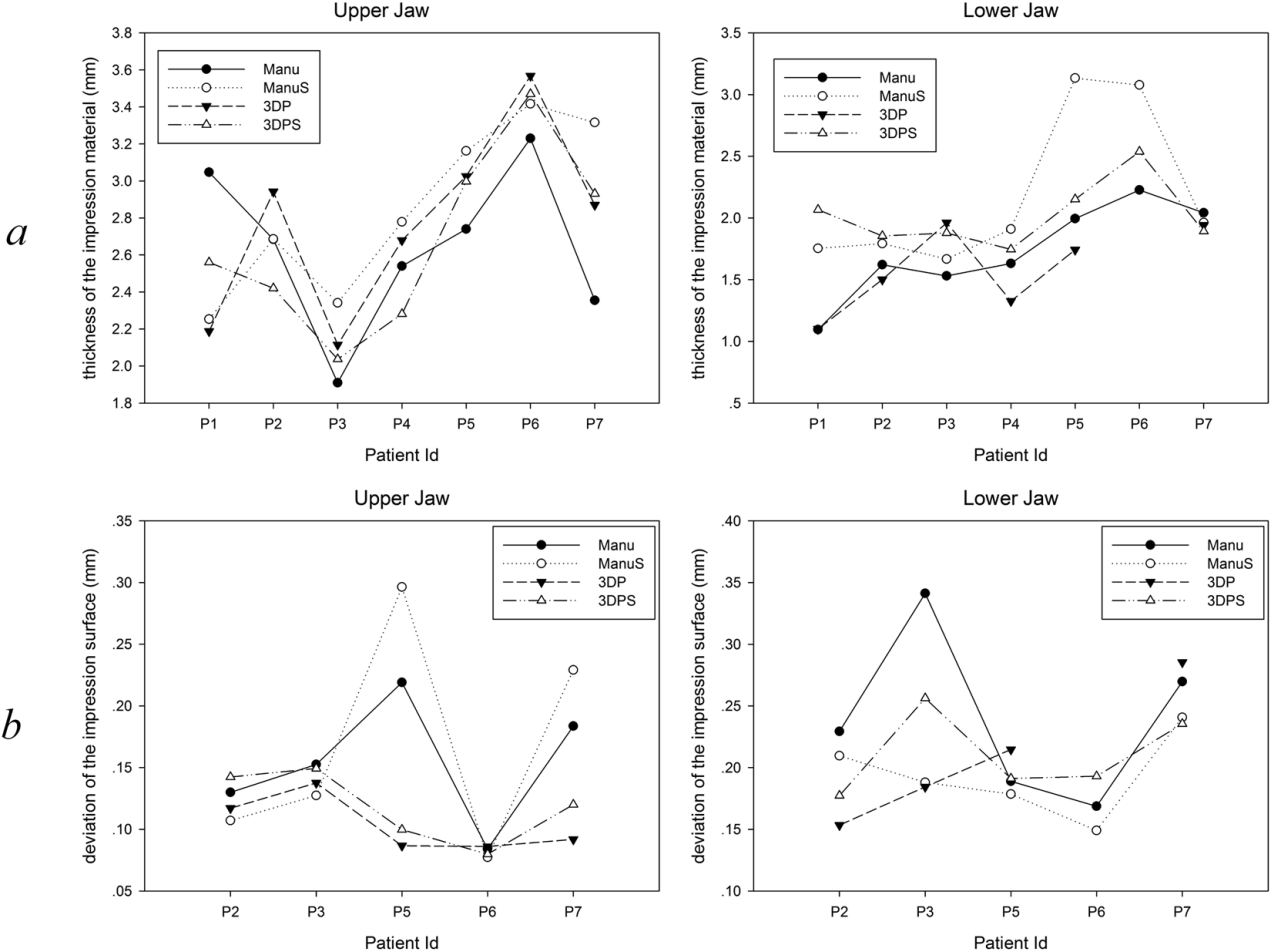
(**a**) Thicknesses of the final impression materials of the four trays and (**b**) the three-dimensional (3D) deviation of the impression surface. Manu = manual tray without tissue stop, ManuS = manual tray with a tissue stop, 3DP = 3D printed tray without a tissue stop; 3DPS = 3D printed tray with a tissue stop

### Data Availability

The datasets generated during and/or analysed during the current study are available from the corresponding author on reasonable request.

## Results

The 3DP trays had similar 3D morphology structure as the manual trays (Fig. 1). Figure 2 demonstrates the 3D analysis of the thickness of a patient’s mandibular impression. The trays with tissue stops (Fig. 2Ab and d) obtained the impression materials with relatively even thickness, which was close to 2 mm. However, the thicknesses of the impression materials from trays without a tissue stops appeared inconsistent in each area (Fig. 2Aa, and c).

The thicknesses of the impression materials from the four different trays for seven patients are shown in Fig. 3. Patient P6’s data were lost because of an accident during the scanning process in which the mounted tray dropped from the platform and the impression became damaged. There was significant individual difference in the thickness of the maxilla tray, which was primarily 2 mm higher than the ideal value.

**Figure 4:**
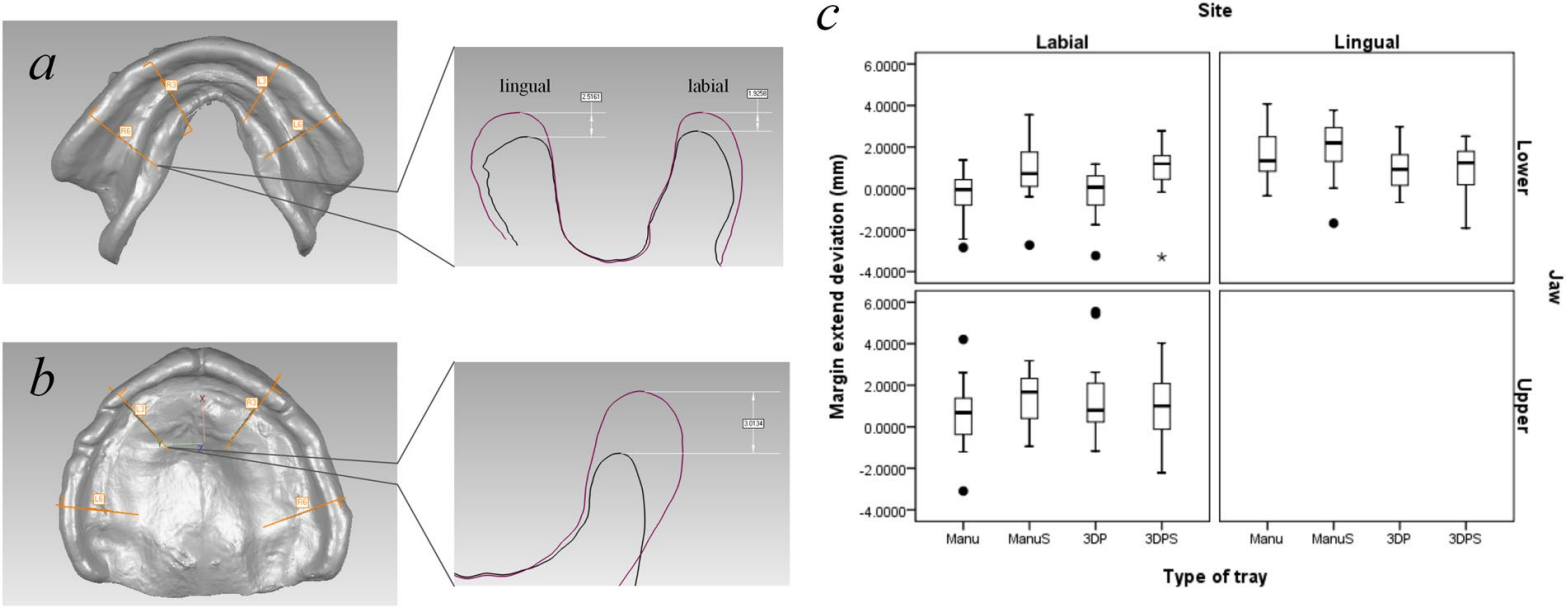
Bisection of the impression from the canine teeth and the first molars section to examine the flange extension. (**a**) The mandible. (**b**) The maxilla (R6 = right first molar, R3 = right canine, L3 = left canine, L6 =  left first molar; the red line is the outline of the reference impression and the black line is the outline of the impression to be tested). (**c**) The data of the deviation of the flange extension between all the final impressions and the reference impressions. The filled circle (●) indicates an abnormal value and the star (★) indicates an extremely abnormal value.

By contrast, the thickness of the mandibular impression material was generally stable at 2 mm. Of these the impressions, the thickness of the impression material from 3DPS tray was the closest to 2 mm. The deviation of the impression morphology in non-marginal areas from different trays is shown in Fig. 3b. The average deviations of the maxilla and mandible were both within 0.5 mm.

Further statistical analysis showed that the impression thickness deviation from the maxilla 3DP tray was less than that of the manual tray (P = 0.008, power = 0.892) (Table 1). The thickness of the impression material from the maxilla 3DPS tray was similarly superior to that of the ManuS tray (P = 0.031, power = 0.647). The difference was statistically significant.

**Table 1:** Results of paired Student’s *t* test of the impression material thickness deviation and three-dimensional morphology deviation from four different trays.

Pair	Upper jaw			Lower jaw		
	Differences (mean ± SD/mm)	p	Power	Differences (mean ± SD/mm)	p	Power
d_Manu_−d_ManuS_	0.04 ± 0.31	0.733	0.060	0.30 ± 0.25	0.020*	0.754
d_3DP_−d_3DPS_	0.06 ± 0.27	0.584	0.079	0.43 ± 0.45	0.067	0.563
d_Manu_−d_3DP_	0.35 ± 0.24	0.008**	0.892^#^	0.36 ± 0.20	0.006**	0.975^#^
d_ManuS_−d_3DPS_	0.37 ± 0.35	0.031*	0.647	0.52 ± 0.38	0.012*	0.852^#^
E_Manu_−E_ManuS_	−0.014 ± 0.046	0.541	0.083	0.046 ± 0.060	0.159	0.263
E_3DP_−E_3DPS_	−0.014 ± 0.014	0.077	0.401	−0.006 ± 0.054	0.847	0.054
E_Manu_−E_3DP_	0.050 ± 0.059	0.13	0.308	0.048 ± 0.086	0.346	0.163
E_ManuS_−E_3DPS_	0.049 ± 0.100	0.334	0.137	−0.018 ± 0.040	0.379	0.123

*P < 0.05, **P < 0.01, ^#^power > 0.8.

‘d’ represents the impression thickness deviation.

‘E’ represents the 3-D morphology deviation.

3DP, three-dimensional custom-made tray without a saddle-shaped tissue stop; 3DPS, three-dimensional custom-made tray with a saddle-shaped tissue stop; Manu, manufactured manual tray; ManuS, manufactured manual tray with tissue stop.

The closeness of the thickness of the mandibular impression material to the ideal value were ManuS > Manu (P = 0.020, power = 0.754), 3DP > Manu (P = 0.006, power = 0.975), and 3DPS > ManuS (P = 0.012, power = 0.852). The three-dimensional deviations of the impression surfaces from different trays were not statistically significant (Table 1).

The flange extensions of the final impressions from different trays are shown in Fig. 4c. They were sorted, based on the tray type, the position of the maxilla, mandible, and buccal, lingual. The box chart created by SPSS (SPSS Inc.) shows nine abnormal values within all the 232 data points. As Fig. 4c shows, the extension range of the labial-buccal border in the final impression from the trays (including manually and digitally made trays) was close to the reference impression, and the flange extensions tended to be insufficient in the rest of the groups (within approximately 1–2 mm). However, insufficient flange extension was more apparent in trays with tissue stops than in trays without tissue stops.

## Discussion

This study adopted three-dimensional scanning and measuring technology in calculating the thickness of the impression material, and the point-cloud data of the tissue surface of the trays are obtained by using the dental model scanner. However, based on the principle of point-cloud generation from an optical scanner, these point clouds were not evenly distributed, but were intense in an area with an abruptly changing curvature. To ensure the equality of distribution, the point-cloud of the tissue surface of the tray was screened with equal spacing. The distance from the screened points to the triangular meshing surface of the impression surface was measured, which represents the average thickness of the impression material. During the analysis process, we effectively eliminated the influence of the patients’ individual differences by matching the data of the impressions from the same patient’s corresponding trays.

The mandibular alveolar ridge of edentulous patients has a ‘u’ shape and slopes steeply from the alveolar ridge crest to the buccal-labial or the lingual side, which tend to help placing a saddle type tissue stop in the correct position. By contrast, the slope of the palatal side of maxillary alveolar ridge is relatively gentle and cannot resist the forward offset of the tray. Once the offset happens, the tissue stop may cause a premature contact point or pressure point, which will prevent correct placement of the tray. Moreover, because of the large area of the maxilla, the pressure from the maxillary impression material on the tray is larger than that of the mandible under the same intensity of pressure. Therefore, it requires greater pressure to press the tray into the right place of the maxilla. This factor may explain why the impression material of the maxillary tray is generally thicker.

Many clinical factors such as the humidity of the mouth^21^, change in the mandibular morphology with mouth opening^22,23^, flabby alveolar ridge^24,25^ and a patient’s cooperation with the dentist^26,27^ affect the precision of an impression. In this study, we enrolled only patients who had favourable characteristics such as non-heavily absorbed alveolar bone, good cooperation with doctors, and healthy oral mucosa to avoid adverse effects in the experiment. However, in the general clinic situation, a flat and low alveolar ridge is unable to effectively limit the position of the tissue stop, which will consequently influence the location of 3DPS tray and ManuS tray. Concerning the aforementioned factors, when applying a saddle-shaped tissue stop to the maxillary edentulous jaw or if a patient has an alveolar ridge with poor condition, or patient has poor cooperation, problems may arise in the accuracy and reliability in the placement of the tray, which should remind clinicians to improve the design of the tissue stop.

The experimental results showed that the thickness of the impressions from digital trays is superior to that of manual trays (i.e. 3DP > Manu and 3DPS > ManuS) in the maxilla and the mandible. In view of the limited sample size, the statistic power must be considered. We believe a power > 0.8 is sufficient (Table 1). Digitally designed trays reserve more homogeneous spaces for the impression materials than manual trays^10^, which guarantees the precision of the impressions^9,11^. The manufacture process and manual operation of digital trays is superior; therefore, they appear to be a very promising substitute for manual edentulous trays in the future.

With respect to the influence on deviation of the thickness of impression material caused by tissue stop, statistical differences were only investigated between mandibular ManuS tray and Manu tray. However, the statistic power (0.754) was slightly lower than 0.8. This finding is probably because of the small sample size, which may be insufficient to detect differences between the two tray types.

The use of a tissue stop is helpful for the correct placement of trays, thus avoiding flange overextension. However, the experimental results also indicated that if the trays were not border-molded beforehand, there could be insufficient flange extension in the final impressions. This finding was more obvious in trays with tissue stops, and must be avoided by intensifying the border-molding or by improving the tray morphology. Moreover, if a doctor stretches the patient’s mouth and lip muscles excessively during the passive border-molding step in the process of making the primary impression, then excessive border-molding is likely to occur and result in the lack of flange extension. For the upper jaw, if the impression material is too viscous with poor fluidity, which cannot fully fill in the space, or if the material is too thin with high liquidity, which flows down owing to gravity, then less flange extension will occur. In these situations, the border-molding of the custom tray with border-molding material in advance of the impression-taking will provide effective support of the impression material in the marginal area, and thus will facilitate adequate flange extension.

Moreover, as an artificial ‘pressure area’, a tissue stop area may deform local mucosal tissue, which will consequently affect the accuracy of the impressions. This study compared the impressions from different types of trays with border-bolded 3DP trays. All average deviations were within 0.5 mm. As previously mentioned, there was no evidence proving that a tissue stop influences the quality of impressions. However, this study does not exclude the possibility of differences as there are only a small number of samples. What’s more, impressions from border-molded 3DP trays were not necessarily the most accurate.

From this study, we concluded that (1) the digitally designed and the 3D-printed custom trays had better adaptations since impressions made using these trays had better thickness distribution than those obtained with manually made trays and (2) regardless of whether there was a tissue stop design, insufficient flange extensions occurred when the digital trays or manual trays were not pre-border-molded. Further studies will need to focus on improving the 3D design of the trays and tissue stops to facilitate the border-molding procedure. In addition, the influence of better designed digital trays on the quality of impressions and the adaptability of the final denture will be investigated in future studies.
